# Ischemia-Reperfusion Injury in a Simulated Lung Transplant Setting Differentially Regulates Transcriptomic Profiles between Human Lung Endothelial and Epithelial Cells

**DOI:** 10.3390/cells10102713

**Published:** 2021-10-10

**Authors:** Gaowa Saren, Aaron Wong, Yun-Bi Lu, Cristina Baciu, Wenyong Zhou, Ricardo Zamel, Sahar Soltanieh, Junichi Sugihara, Mingyao Liu

**Affiliations:** 1 Latner Thoracic Surgery Research Laboratories, Toronto General Hospital Research Institute, University Health Network, Toronto, ON M5G 1L7, Canada; srgw123@126.com (G.S.); aaron.wong@uhnresearch.ca (A.W.); yunbi@zju.edu.cn (Y.-B.L.); Cristina.Baciu@uhnresearch.ca (C.B.); dr.zhouwenyong@shchest.org (W.Z.); ricardo@zamel.ca (R.Z.); ssoltanieh@immunit.com (S.S.); jsugihar@g.ucla.edu (J.S.); 2 Institute of Medical Science, Temerty Faculty of Medicine, University of Toronto, Toronto, ON M5S 1X8, Canada; 3 Department of Pharmacology, School of Medicine, Zhejiang University, Hangzhou 310058, China; 4 Department of Surgery, Medicine and Physiology, Temerty Faculty of Medicine, University of Toronto, Toronto, ON M5S 1X8, Canada

**Keywords:** ischemia-reperfusion injury, cell culture model for lung transplantation, transcriptomics and bioinformatics, functional genomics, translational research

## Abstract

Current understanding of mechanisms of ischemia-reperfusion-induced lung injury during lung preservation and transplantation is mainly based on clinical observations and animal studies. Herein, we used cell and systems biology approaches to explore these mechanisms at transcriptomics levels, especially by focusing on the differences between human lung endothelial and epithelial cells, which are crucial for maintaining essential lung structure and function. Human pulmonary microvascular endothelial cells and human lung epithelial cells were cultured to confluent, subjected to different cold ischemic times (CIT) to mimic static cold storage with preservation solution, and then subjected to warm reperfusion with a serum containing culture medium to simulate lung transplantation. Cell morphology, viability, and transcriptomic profiles were studied. Ischemia-reperfusion injury induced a CIT time-dependent cell death, which was associated with dramatic changes in gene expression. Under normal control conditions, endothelial cells showed gene clusters enriched in the vascular process and inflammation, while epithelial cells showed gene clusters enriched in protein biosynthesis and metabolism. CIT 6 h alone or after reperfusion had little effect on these phenotypic characteristics. After CIT 18 h, protein-biosynthesis-related gene clusters disappeared in epithelial cells; after reperfusion, metabolism-related gene clusters in epithelial cells and multiple gene clusters in the endothelial cells also disappeared. Human pulmonary endothelial and epithelial cells have distinct phenotypic transcriptomic signatures. Severe cellular injury reduces these gene expression signatures in a cell-type-dependent manner. Therapeutics that preserve these transcriptomic signatures may represent new treatment to prevent acute lung injury during lung transplantation.

## 1. Introduction

Lung transplantation (LTx) is a therapeutic option for patients with end-stage lung disease. However, primary graft dysfunction is an important complication, contributed by ischemia-reperfusion injury (IRI). Understanding the cellular and molecular mechanisms of IRI in LTx is crucial.

Multiple genes related to acute inflammation and cell death are enriched in human lung grafts during reperfusion compared with hypothermic preserved donor lungs [1]. These results confirm the previous reports on upregulation of genes in Toll-like receptor pathways and cytokines [2], activation of MAP kinases [3], and increased apoptosis [4] in human lungs. Intracellular signal transduction pathways related to inflammation and cell death could be the therapeutic targets [5]. The lung contains more than 40 different cell types; single-cell RNA sequencing has been developed to explore cell-type-specific gene expression profiles [6]. The development and application of cell and systems biology may significantly advance LTx research.

To better understand the cellular and molecular mechanisms of IRI in LTx, human lung cell culture models have been developed. Human lung cancer A549 cells were first used to simulate static cold storage (SCS) and warm reperfusion after transplantation [7]. This model was further optimized using normal human lung epithelial BEAS-2B cells; the SCS and warm reperfusion conditions induced acute inflammatory response, apoptosis, and regulated necrosis, which is mediated through protein kinase Cδ activation [8]. Blocking protein kinase Cδ with a specific peptide inhibitor prevented IRI in rat lung transplant models [8,9]. In this cell culture model, ischemia-reperfusion (IR) also induced necroptosis [10]. Blocking necroptosis prevented and reduced IRI in rat lung transplants [11]. 

Moreover, using the cell culture model, it was found that alpha-1 antitrypsin (A1AT), a well-known neutrophil elastase inhibitor, has anti-inflammatory and anti-cell death function, which was further validated through rat lung transplant [12] and pig lung transplant [13] models. Ex vivo lung perfusion (EVLP) is a new technology for donor lung assessment and repair [14]. During EVLP, A1AT reduced IRI in pig lungs [15] and repaired severely damaged human donor lungs declined by the clinical transplant program [16]. This series of studies presented a drug discovery pipeline to translate from basic scientific research to clinical application [17] and to support the cell culture model as a useful tool for initial drug screening and exploration of underlying mechanisms.

Casiraghi et al. used human umbilical vein endothelial cells to simulate hypothermic preservation and warm reperfusion [18]. A human pulmonary microvascular endothelial cell line was established by Kirkpatrick et al. [19], which is more relevant to lung biology research. In this study, we used this cell line to further develop the cell culture model for lung transplant research.

The function and phenotypic features of endothelial and epithelial cells are distinct, and these cells may respond to the same IR condition differently. We hypothesized that pulmonary endothelial and epithelial cells have distinct gene expression profiles, which determine the phenotypic characteristics and function of each cell type and determine the cellular responses to different cellular stress, especially cold ischemia–warm reperfusion-induced cellular responses, and IRI in LTx.

Using microarray and transcriptomic analyses, we compared the signatures between these two cell types. We then compared the changes of these phenotypic characteristics under different IR conditions. 

## 2. Materials and Methods

### 2.1. Cell Lines and Reagents

Normal human bronchial epithelial cell line (BEAS-2B) was obtained from ATCC (Manassas, VA, USA), and cells were cultured in low glucose Dulbecco’s modified Eagle’s medium (DMEM) supplemented with 10% fetal bovine serum (FBS, GIBCO, Carlsbad, CA, USA), as described before [8,10]. A human pulmonary microvascular endothelial cell line (HPMEC) was obtained from Kirkpatrick’s research lab [19] and cultured on plates precoated with 0.2% gelatin. The culture medium used was medium M199 supplemented with 20% FBS, Glutamax (2 mM), endothelial cell growth supplement from bovine neural tissue (ECGS 50 μg/mL, Sigma Aldrich), heparin (50 U/mL), and Penicillin–Streptomycin solution (100 U/100 μg/mL). Cells were cultured at 37 °C with 5% CO_2_ until confluent. The medium was replaced every 2–3 days. Subcultures were obtained by trypsin/EDTA treatment of confluent monolayer cells, and then cells were resuspended in a fresh culture medium. 

Cell differentiation phenotypes were characterized by immunofluorescence staining of endothelial cell marker vWF, by Western blotting of endothelial cell markers (vWF and VE-cadherin) and epithelial cell markers (XB130 and E-cadherin), and by flow cytometry of endothelial cell marker PECAM-1.

### 2.2. Western Blotting

Cells were washed with ice-cold PBS twice and lyzed at 4 °C with RIPA buffer (VWR, Radnor, PA, USA). Cellular protein was extracted after centrifugation at 10,000× *g* at 4 °C for 20 min. The protein content was determined using the Pierce™ BCA Protein Assay Kit (Thermo Fisher Scientific, Waltham, MA, USA). Samples (40 μg protein each) were loaded on a 10% SDS–PAGE gel. The proteins were transferred onto a PVDF membrane (Bio-Rad). The membrane was incubated overnight at 4 °C with a specific primary antibody diluted in 5% bovine serum albumin and then blocked in 5% fat-free milk in TBST at room temperature for 1 h. The horseradish peroxidase-conjugated secondary antibody (Invitrogen, Carlsbad, CA, USA) was used for incubation at room temperature for 2 h, and the membrane was washed with TBST three times. Detection was carried out by incubating the membrane with a chemiluminescent reagent. 

### 2.3. Flow Cytometry

Cells were detached using a trypsin–EDTA solution and centrifugated at 1000× *g* at 4 °C. The cells were resuspended in diluent (PBS containing 1% bovine serum albumin) and adjust cell suspension to 1 × 10^6^ cells per tube. Then, 5 μL of primary antibody (mAb against PECAM-1, Sigma, St. Louis, MO, USA) was added to each tube at room temperature for 30 min. For washing the cells, 2 mL diluent was added to each tube, and cell pellets were obtained by centrifugation at 500× *g* for 10 min. The washing procedure was repeated three times. After removing the supernatant, cells were resuspended in 100 μL of FITC-conjugated secondary antibody (1:200) for 30 min at room temperature. The CytoFLEX LX flow cytometer (Beckman Coulter, Indianapolis, IN, USA) was used to determine the number of PECAM-1 positive cells.

The percentage of apoptotic and necrotic cell death was determined through FAM-VAD-FAM caspase 3/7 immuno-fluorescent staining (Immunochemistry, Bloomington, MN, USA) and PI staining (Invitrogen, Carlsbad, CA, USA) followed by flow cytometry [8].

### 2.4. Simulated IR Model for Lung Transplantation

A cell culture model that similar hypothermic preservation and warm reperfusion of LTx [7,8,10] was further developed for both endothelial and epithelial cells. Briefly, cold ischemia was simulated by replacing cell culture medium with cold Perfadex^®^ solution (Medisan Pharmaceuticals, Uppsala, Sweden), a clinically used solution for donor lung preservation, and stored in a 4 °C chamber for 6 h, 12 h, 18 h, 24 h. Clinically, donor lungs are inflated with 50% O_2_ during hypothermic preservation; thus, the chamber was filled with room air mixed with 50% O_2_. To simulate reperfusion, the cold Perfadex^®^ solution was changed back to a warm serum-containing culture medium, and cells were further cultured at 37 °C for 2 h or 4 h. The cell morphology was recorded using a phase-contrast microscope. Cell viability was determined by trypan blue dye exclusion assay [8]. 

### 2.5. Gene Expression

Total RNA was purified using RNeasy Mini Kit (QIAGEN; Hilden, Germany). RNA quality was tested using a NanoDrop spectrophotometer (VWR) and Bioanalyzer (Agilent; Santa Clara, CA, USA). Gene expression profiles were measured using Affymetrix Human Gene 2.0 ST Array according to manufacturer protocols at the Princess Margaret Genomics Center (Toronto, ON, Canada). Gene expression was normalized using a Robust multi-array average [20]. Annotations were conducted using Brainarray version 19 in the “oligo” R package [21]. Differential gene expression, principal component analysis, hierarchical clustering, and Venn Diagrams were performed in R with various packages [21]. Differential gene expression (DGE) was defined by a cut-off adjusted *p* value (*p* < 0.05) and a false discovery rate (FDR) < 0.05. Original data are available from the Gene Expression Omnibus database (GSE172222).

### 2.6. Gene Set Enrichment Analysis (GSEA)

Ranked gene lists were generated by scoring each gene using the formula: Gene Score = −ln (*p* value from DGE) * (Sign of Gene Fold Change). These pre-ranked datasets were used as the input for GSEA. Briefly, gene sets that met an FDR < 0.05 were visualized in EnrichmentMap and clustered using AutoAnnotate in the Cytoscape software package, as described in Reidmand et al. [22]. Clusters with four or more gene sets were chosen for detailed analysis.

## 3. Results

### 3.1. Cold Preservation and Warm Reperfusion Reduced Viability of HPMEC and BEAS-2B Cells

**Figure 1:**
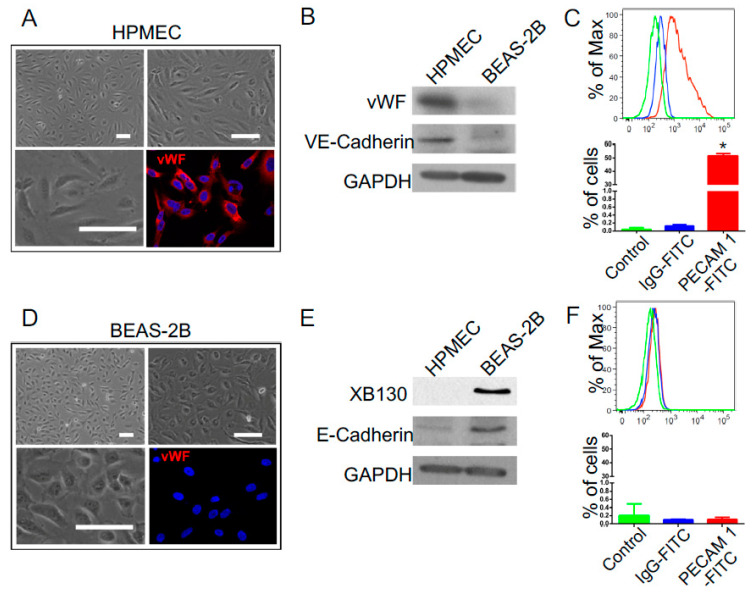
Phenotypic characterization of human pulmonary microvascular endothelial cells (HPMECs) and human lung epithelial BEAS-2B cells: (**A**) HPMEC cells exhibited spindle shape and positive immunofluorescent staining with vWF (red) (scale bar = 50 μm); (**B**) the expression of endothelial markers, vWF, and VE-cadherin was detected by Western blotting in HPMEC cells but not in BEAS-2B cells. GAPDH was used as housekeeping control; (**C**) another endothelial marker, PECAM-1, was detected positively in HPMEC cells by flow cytometry; * *p* < 0.05 vs. other two groups. (**D**) BEAS-2B cells exhibited cuboidal shape, and vWF staining was negative (scale bar = 50 μm); (**E**) the expression of epithelial markers, XB130 and E-cadherin, was detected by Western blotting in BEAS-2B cells but not in HPMEC cells; (**F**) PECAM-1 was negative in BEAS-2B cells, as analyzed by flow cytometry.

Under normal culture conditions, confluent HPMEC cells showed spindle-like morphology and were positively stained with endothelial cell marker, vWF (Figure 1A). Cell surface marker VE-cadherin was detected by Western blotting (Figure 1B). Expression of another endothelial marker PECAM1 was detected with flow cytometry (Figure 1C). By contrast, BEAS-2B cells showed cuboidal shape (Figure 1D), and surface marker E-cadherin was detected by Western blotting (Figure 1E). XB130, an adaptor protein that is involved in the regulation of cell proliferation and motility in BEAS-2B cells [23,24,25], was only observed in lung epithelial cells (Figure 1E), whereas vWF and PECAM1 were not detected from epithelial cells (Figure 1D–F).

**Figure 2:**
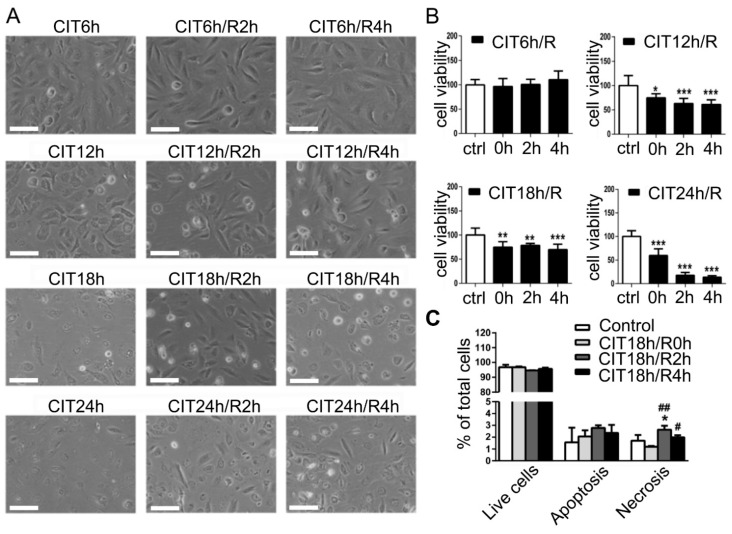
Hypothermic ischemia and normothermic reperfusion induced changes in cell morphology and reduced cell viability in human pulmonary microvascular endothelial cells (HPMECs): (**A**) HPMEC cells underwent 6, 12, 18, or 24 h CIT, followed by 2 or 4 h warm reperfusion. A CIT time-dependent change of cell morphology and loss of cells were observed, which was increased after reperfusion (scale bar = 50 μm); (**B**) cell viability was quantified via trypan blue exclusion assay. Reduced cell viability was observed after 12 h CIT and reperfusion, which was further enhanced after 18 h or 24 h CIT. * *p* < 0.05, ** *p* < 0.01, *** *p* <0.001 vs. control conditions; (**C**) HPMECs were double stained with FAM-VAD-FAM caspase 3/7 immunofluorescence and PI and then analyzed via flow cytometry to quantify apoptotic and necrotic cell death. * *p* < 0.05 vs. control; # *p* < 0.05, ## *p* < 0.01 vs. CIT 18 h group.

Clinically, human donor lungs are preserved on ice within 6–8 h for storage and transportation. Prolonged preservation enhances IRI in lung transplants; thus, it is a major area of research in organ preservation and transplantation. To simulate cold preservation, we switched HPMEC or BEAS-2B cells from regular culture condition (37 °C, 5% CO_2_, in serum-containing culture medium) to SCS condition (4 °C, 50% O_2_ in lung preservation solution). From 6 h to 24 h, SCS condition changed cell morphology and reduced cell numbers in a time-dependent manner, which was further amplified after warm reperfusion in HPMEC cells (Figure 2A). Cell viability significantly reduced after 12 h CIT and further decreased after 18 h and 24 h CIT, and especially after reperfusion (Figure 2B). Flow cytometry demonstrated that after 18 h CIT, necrosis increased after 2 or 4 h reperfusion (Figure 2C). Similarly, as seen in HPMEC cells and as we reported previously [8], a CIT time-dependent change of cell morphology and reduction of viability were also observed in BEAS-2B cells (Figure S1). A CIT time-dependent dynamic change in apoptotic and necrotic cell death has been reported in a rat lung preservation/transplant model, which correlated with the severity of IRI after LTx. In that study, significant cell death was also observed after 18 or 24 h CIT [26].

### 3.2. HPMEC and BEAS-2B Cells Showed Significantly Different Transcriptomic Gene Profiles

**Figure 3:**
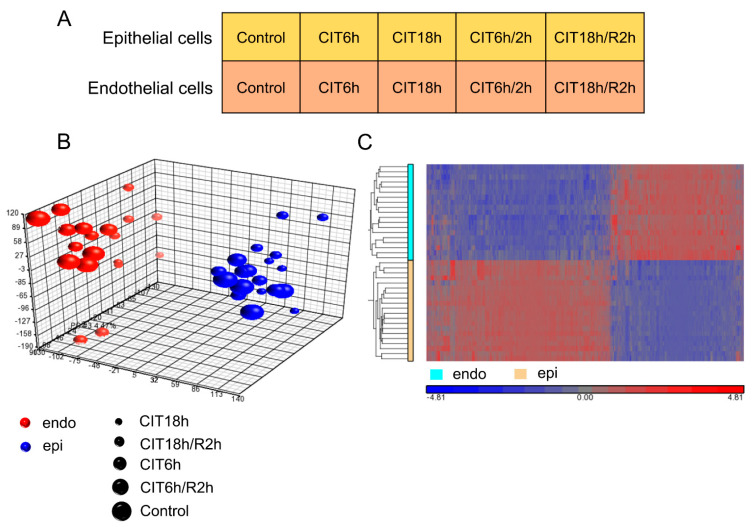
Hypothermic ischemia and normothermic reperfusion-induced gene expression profiles in human pulmonary microvascular endothelial cells (HPMECs) and in human lung epithelial BEAS-2B cells: (**A**) experimental design. Both HPMEC cells and BEAS-2B cells underwent 6 or 18 h CIT, followed by 2 h warm reperfusion (CIT/R); (**B**) principal component analysis (PCA) demonstrated that the overall gene expression of HPMEC cells and BEAS-2B cells were clearly distinct from each other; (**C**) hierarchical clustering analysis demonstrated that differentially expressed genes were also different between HPMEC cells and BEAS-2B cells.

To determine the transcriptomic differences between human lung endothelial and epithelial cells, RNA samples were collected from HPMEC and BEAS-2B cells after normal culture as control, or after 6 h/18 h CIT, or 2 h reperfusion after 6 h/18 h CIT (Figure 3A) and processed for microarray studies. Principle component analysis showed that the overall gene expression profiles were primarily separated between HPMEC and BEAS-2B cells, and within the same cell type, 18 h CIT alone or 2 h reperfusion after 18 h CIT further affected the overall gene expression profiles (Figure 3B). A heatmap showed that differentially expressed genes (FDR, *p* < 0.05, FC > |1.3|) were also different between the endothelial and epithelial cells (Figure 3C). Understanding these basic differences are essential for the cellular and molecular mechanisms in lung biology. In this study, we focused on the phenotypic differences between these two cell types and how IR conditions affect them.

Under control conditions, of the 25,582 gene transcripts analyzed, there were 9900 differentially expressed (DE) genes between endothelial and epithelial cells at FDR < 0.05. GSEA analysis identified 331 enriched gene sets between these two cell types, of which 123 were enriched in endothelial cells, and 208 were enriched in epithelial cells. 

**Figure 4:**
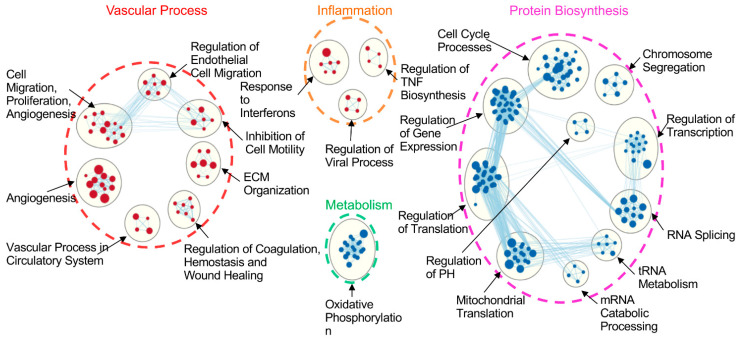
Enriched clusters of differentially expressed (DE) gene sets between human lung endothelial and epithelial cells. GSEA assay showed DE gene sets (FDR < 0.05) between HPMEC and BEAS-2B cells. Gene clusters enriched in endothelial cells are shown as red nodes, which mainly fell into two themes: vascular process and inflammation. Gene clusters enriched in epithelial cells are shown as blue nodes, which were dominant in protein biosynthesis and metabolism.

These gene sets were further grouped into 21 gene clusters (Figure 4). Of those, 10 gene clusters were dominant in endothelial cells and mainly involved in the vascular process (such as genes related to cell migration, proliferation, angiogenesis, vascular process, coagulation, ECM organization) and inflammation (such as responses to interferons, regulation of TNF biosynthesis). There were 11 gene clusters dominant in epithelial cells, mainly associated with protein biosynthesis (e.g., regulation of gene expression, regulation of transcription, RNA splicing, regulation of translation) and metabolism (e.g., oxidative phosphorylation) (Figure 4).

### 3.3. IR Differentially Affected Gene Expression in Human Pulmonary Endothelial and Epithelial Cells in a CIT Time-Dependent Manner 

We then examined the DE genes of each cell type after CIT and reperfusion. For endothelial cells, 5421 DE genes were identified by ANOVA and 2957 DE genes by p-adjusted Tukey HSD test. PCA showed that reperfusion groups (C6R2 and C18R2) were separated from the other three groups (Figure S2A). Among the 5 groups, there were 10 possible comparisons, of which 306 genes were differentially expressed in at least 6 group comparisons. Unsupervised clustering heatmap showed that the control and CIT 6 h and CIT 18 h groups were grouped together, separated from C6R2 and C18R2 groups (Figure S2B). For epithelial cells, 5234 DE genes were identified by ANOVA, 2901 by Tukey HSD test, and 364 were in at least 6 group comparisons. The PCA and heatmap showed similar separation between control/CIT groups and reperfusion groups (Figure S3). These results indicate that at 4 °C mRNA levels remained relatively stable; it was the reperfusion that activated gene expression and had a significant impact on mRNA levels.

### 3.4. IR-Induced Loss of Phenotypic Gene Expression Characteristics of Human Lung Endothelial and Epithelial Cells 

We then focused on the effects of IR on the phenotypic differences observed between human lung endothelial and epithelial cells. At the FDR < 0.05 level, the numbers of DE genes between these two cell types remained at similar levels, after different periods of CIT and reperfusion (Figure S4A). Venn diagram shows these DE genes were heavily overlapped among all groups. A total of 6703 genes were differentially expressed in all 5 groups, and under each experimental condition, hundreds of unique DE genes could be found between these two cell types (Figure S4B). We then used GSEA to identify enriched gene sets between two cell types. At FDR < 0.05 level, approximately 300 to 400 gene sets were found in control, CIT 6 h and CIT 6 h plus reperfusion groups, of which the numbers of enriched gene sets in endothelial cells were less than that in the epithelial cells (Figure S4C). However, after 18 h CIT, GSEA only identified 160 enriched gene sets; among them, only 18 gene sets were expressed in epithelial cells. After 2 h reperfusion, only 83 gene sets were left in endothelial cells and none in epithelial cells (Figure S4C). These results indicate the phonotypic transcriptomic signatures between endothelial and epithelial cells are reduced when cells are undergoing prolonged preservation and reperfusion, especially in epithelial cells.

### 3.5. Prolonged CIT Dramatically Changed the Cell-Type Specific Signatures

Similar to the results of the control group (Figure 4), there were 22 enriched gene clusters in the CIT 6 h group, of which each cell type had 11 clusters (Figure S5). The functions of these clusters were very similar to that in the control group; gene sets in each cluster were also very similar to that in the control group. After CIT 6 h and 2 h reperfusion, 23 clusters were identified, of which 10 were dominant in endothelial cells, and 13 in epithelial cells (Figure S6). Again, the functions of these clusters were very similar to those found in the control (Figure 4) and CIT 6 h groups (Figure S5).

**Figure 5:**
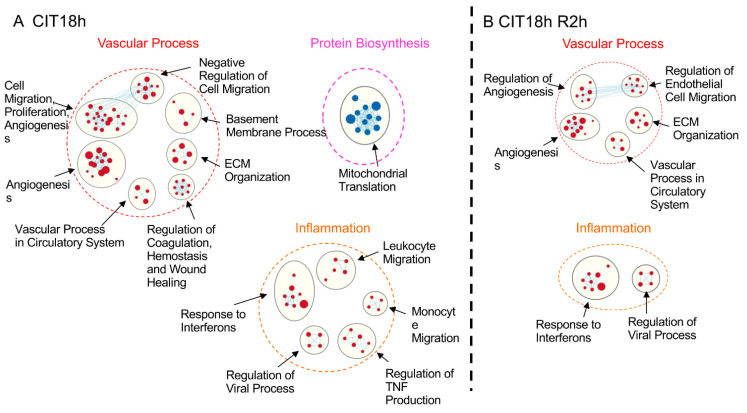
CIT 18 h and CIT 18 h, followed by 2 h reperfusion, reduced enriched DE gene clusters between endothelial and epithelial cells dramatically: (**A**) after 18 h CIT, only one DE gene cluster was left in epithelial cells (blue nodes), and the numbers of gene sets in endothelial cells were reduced (red nodes); (**B**) after 18 h CIT and 2 h reperfusion, no epithelial-cell-specific gene cluster was found, and the numbers of gene clusters and gene sets in each cluster of endothelial cells were reduced. The cut-off of DE gene-set clusters was set at FDR < 0.05.

After CIT 18 h, however, only 13 gene clusters were left, of which 12 were dominant in endothelial cells (Figure 5A). The two major cluster themes predominantly in endothelial cells found in the control group (vascular process and inflammation) remained, with similar gene subsets. In epithelial cells, the only dominant cluster left was that of mitochondrial translation (Figure 5A). After 2 h reperfusion, only 7 gene clusters in endothelial cells remained (Figure 5B). These results indicate that the IRI significantly altered the phenotypic features in lung cells, especially in epithelial cells.

**Figure 6:**
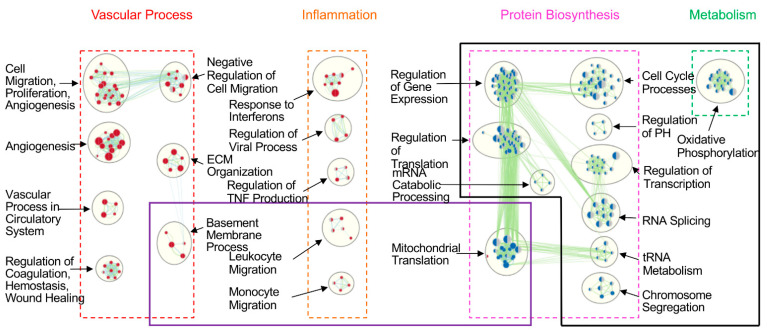
Dynamics of reduction of enriched DE gene clusters between endothelial cells and epithelial cells. Under control conditions, two major themes of enriched gene clusters were found in endothelial cells: vascular process and inflammation (red nodes), and two major themes in epithelial cells: protein biosynthesis and metabolism (blue nodes). The black box showed the loss of enriched gene clusters after CIT 18 h. The purple box showed the further loss of enriched gene clusters after 2 h reperfusion. The cut-off of enriched DE gene clusters is FDR < 0.05.

Comparing differences between endothelial and epithelial cells under control conditions, and after 18 h CIT, epithelial cells lost all the protein-biosynthesis-related gene clusters except that of mitochondrial translation (Figure 6, boxed with black line). In CIT18/R2 group, epithelial cells further lost mitochondrial translation gene cluster. In addition, endothelial cells lost gene clusters related to the basement membrane process, leukocyte migration, and monocyte migration (Figure 6, boxed with purple line).

## 4. Discussion

### 4.1. IR-Induced Cell Death and Gene Expression in Human Lung Endothelial and Epithelial Cells 

Gas exchange is the primary function of the lung; injury of alveolar epithelial and endothelial cells during IR contributes to the development of primary graft dysfunction in LTx [27]. In this study, we further developed the cell culture model that simulates major features of cold preservation and warm reperfusion with human pulmonary microvascular endothelial cells. Similar to what we have previously observed in human lung epithelial cells, simulated SCS and warm reperfusion induced a CIT time-dependent cell death in both cell types. The severity of cell death was comparable between both cell types; however, gene expression profiles changed differently. From a cell biology point of view, it is not surprising that endothelial and epithelial cells show different gene expression profiles and respond to similar cellular stress with different genes. However, from the LTx point of view, this is the first time that we can explore transcriptomic signatures of different cell types under simulated IR conditions. Importantly, many of these changes were also observed in human lung transplant samples [1]. These cell-type-related data may help to interpret clinical data and can be used in the future to understand single-cell RNA sequencing data.

### 4.2. The Phenotypic Comparison between Human Lung Endothelial and Epithelial Cells

The cell-type-specific gene expression prompted us to further compare the transcriptomic signatures between human lung endothelial and epithelial cells. Compared with epithelial cells, microvascular endothelial cells are enriched with gene sets related to angiogenesis, cell migration, and coagulation, well-known functions of vascular processes [28,29]. Moreover, these cells highly expressed genes relevant to the inflammation, indicating HPMECs are important for sensing viral and bacterial infection, recruiting leukocytes, and regulating the local inflammatory responses. Endothelium of blood vessels from arterial, venous, and capillary, with different diameters and from different tissues, may have distinct functions that are controlled by characteristic gene expression profiles. Similar research at transcriptomic levels may help us to understand the biological uniqueness of each sub-type of endothelial cells.

Interestingly, enriched gene clusters in epithelial cells are mainly related to the regulation of gene expression, protein synthesis, and metabolism. Indeed, lung epithelium plays an active role in the metabolism of endogenous mediators and xenobiotic agents, and is capable of regeneration, allowing normal cell turnover and restoration after lung injury [30]. On the other hand, the functions of these gene clusters are not specific to epithelial cells; typical features of epithelial cells, such as cytokeratin or specific alveolar epithelial cell markers (such as surfactant proteins) [31] were not detected. We compared the differences between two cell populations, with unsupervised, unbiased approaches. The genes related to transcriptional/translational regulations and protein biosynthesis appeared to be more distinct between these two cell types.

### 4.3. IRI Reduced Phenotypic Characteristics of Human Lung Cells

In this study, we verified that 6 h CIT, a condition used for clinical donor lung preservation, had limited effects on the phenotypic characteristics of endothelial and epithelial cells, either alone or after reperfusion. Extending the donor lung preservation period is one of the main objectives of lung preservation research. In the rat LTx model, preservation of donor lungs over 18 h led to IRI after transplantation [26]. In this cell model, CIT longer than 18 h also led to significantly reduced cell viability during reperfusion. 

The excessive IR stress may lead to severe cellular damage and disorganization in the regulation of mRNA levels. The numbers of DE genes between the two cell types remain at similar levels; however, the numbers of enriched gene sets significantly decreased. The principle of GSEA is to search for sets of genes that are significantly over-represented in a given list of genes. These gene sets consist of genes that function together in a known biological pathway. Therefore, when IRI occurs, even though the mRNA levels of many genes are differentially expressed, if these genes are not functionally related, they will not be identified by the GSEA analysis. 

Associated with the severe IRI is the loss of phenotypic features in the epithelial cells. Gene clusters enriched in epithelial cells significantly reduced after 18 h CIT and completely disappeared after reperfusion. One possible explanation is that epithelial cells are more sensitive to IRI, in terms of regulations of gene expression. On the other hand, the data we used are the differences between two cell types, not absolute measures. It is possible that genes related to transcriptional regulation and protein synthesis are altered in both cell types in a disorganized manner, and as a result, they no longer were recognized by GSEA as known pathways. 

Severe IRI also reduced gene clusters enriched in endothelial cells. IRI is characterized by pulmonary edema caused by endothelial dysfunction, platelet aggregation, and neutrophil activation, and sequestration. Particularly, at the time of reperfusion, free radicals are produced in the endothelial cells, causing an increase in cell membrane permeability. Therefore, the pulmonary vasculature is an important target for therapeutic interventions in LTx. 

### 4.4. Limitations of the Study 

First, BEAS-2B cells were derived from bronchial epithelial cells and therefore may not fully represent alveolar epithelial cells; however, this is the only non-cancer human lung cell line currently available. On the other hand, as presented in the Introduction Section, BEAS-2B cells have been successfully used for mechanistic studies and drug testing under the simulated IR conditions, and results have been validated in vivo with animal models or damaged human donor lungs declined from clinical transplant program [8,9,10,11,12,13,15,16]. Both BEAS-2B and HPMEC cells are immortalized cell lines. Primary human lung cells could be an alternative option, but the viability of these cells and reproducibility of results are of concern. Human inducible pluripotent stem cells could be used to induce alveolar epithelial cells and other cell types, which should be developed further.

Second, in this model, each cell population was studied individually in a two-dimension culture statically. It does not represent the three-dimensional structure of the alveolar unit. The way cells were exposed to the culture medium and to the cold preservation solution did not simulate the in vivo situation. Moreover, during reperfusion, lung cells were exposed to blood flow that contains blood cells, growth factors, hormones, cytokines, stem cells, and therapeutics. These factors were not captured by the current model. The alveolar unit is composed of epithelial and endothelial cells separated by the basement membrane and ventilated and perfused. These three-dimensional structures and mechanical factors are crucial to maintaining basic cellular functions [32]. Recently, a lung-on-a-chip model has been developed with microfluidics technology, with lung endothelial and epithelial cells co-cultured, cyclically stretched, and perfused, which provides a unique opportunity for lung research [33]. Using this platform, it is possible to develop a “lung-transplant-on-a-chip” model. It may provide more accurate information on the biological process of lung cells and could be used to enhance the molecular studies in LTx.

In conclusion, we further developed a cell culture model that simulates the SCS and warm reperfusion process in LTx setting with human pulmonary microvascular endothelial cells. We demonstrated that the human lung endothelial and epithelial cells have distinct transcriptomic signatures. These phenotypic characteristics are preserved well in conditions similar to current clinical donor lung preservation but significantly altered after prolonged SCS. These dramatic changes in phenotypic features at the transcriptomic levels and IR-induced cell-type-specific gene expression, which is under further investigation, hold the key for the discovery of therapeutic targets.

## Data Availability

Original data are available from the Gene Expression Omnibus database (GSE172222).

## Supplementary Materials

The following are available online at https://www.mdpi.com/article/10.3390/cells10102713/s1, Figure S1: Hypothermic ischemia and normothermic reperfusion induce changes of cell morphology, reduce cell viability in human lung epithelial BEAS-2B cells. Figure S2: Differentially expressed genes between the five groups within the HPMEC cells with one-way ANOVA and Tukey HSD test. Figure S3: Differentially expressed genes between the five groups within the BEAS-2B cells with one-way ANOVA and Tukey HSD test. Figure S4: Prolonged cold preservation and reperfusion reduced enriched DE gene-sets. Figure S5: In comparison with control, CIT 6 h did not significantly affect the enriched DE gene clusters between endothelial and epithelial cells. Figure S6: In comparison with control, CIT 6 h followed by 2 h reperfusion also did not significantly affect the enriched DE gene clusters between endothelial and epithelial cells.
