# Supplementary material for: Ischemia-Reperfusion Injury in a Simulated Lung Transplant Setting Differentially Regulates Transcriptomic Profiles between Human Lung Endothelial and Epithelial Cells
Source: Cells. 2021 Oct 10;10(10):2713. doi: 10.3390/cells10102713 (PMC8534993; doi:10.3390/cells10102713)
Supplement: Supplementary file 1 [file cells-10-02713-s001.zip › cells-1385708-supplementary.pdf]

Ischemia–Reperfusion Injury in a Simulated Lung Transplant Setting Differentially Regulates Transcriptomic Profiles between Human Lung Endothelial and Epithelial Cells

Gaowa Saren[1*], Aaron Wong[1,2*], Yunbi Lu[1,3*], Cristina Baciu[1], Wenyong Zhou[1], Ricardo Zamel[1], Sahar Soltanieh[1], Junichi Sugihara[1], Mingyao Liu[1,2,4]

1. Latner Thoracic Surgery Research Laboratories, Toronto General Hospital Research Institute, University Health Network, Toronto, Ontario, Canada;
2. Institute of Medical Science, Temerty Faculty of Medicine, University of Toronto, Toronto, Ontario, Canada;
3. Department of Pharmacology, School of Medicine, Zhejiang University, Hangzhou, Zhejiang, China;
4. Departments of Surgery, Medicine and Physiology, Temerty Faculty of Medicine, University of Toronto, Toronto, Ontario, Canada
*Equal contribution as the first author
Corresponding author: Mingyao Liu, MD, MSc

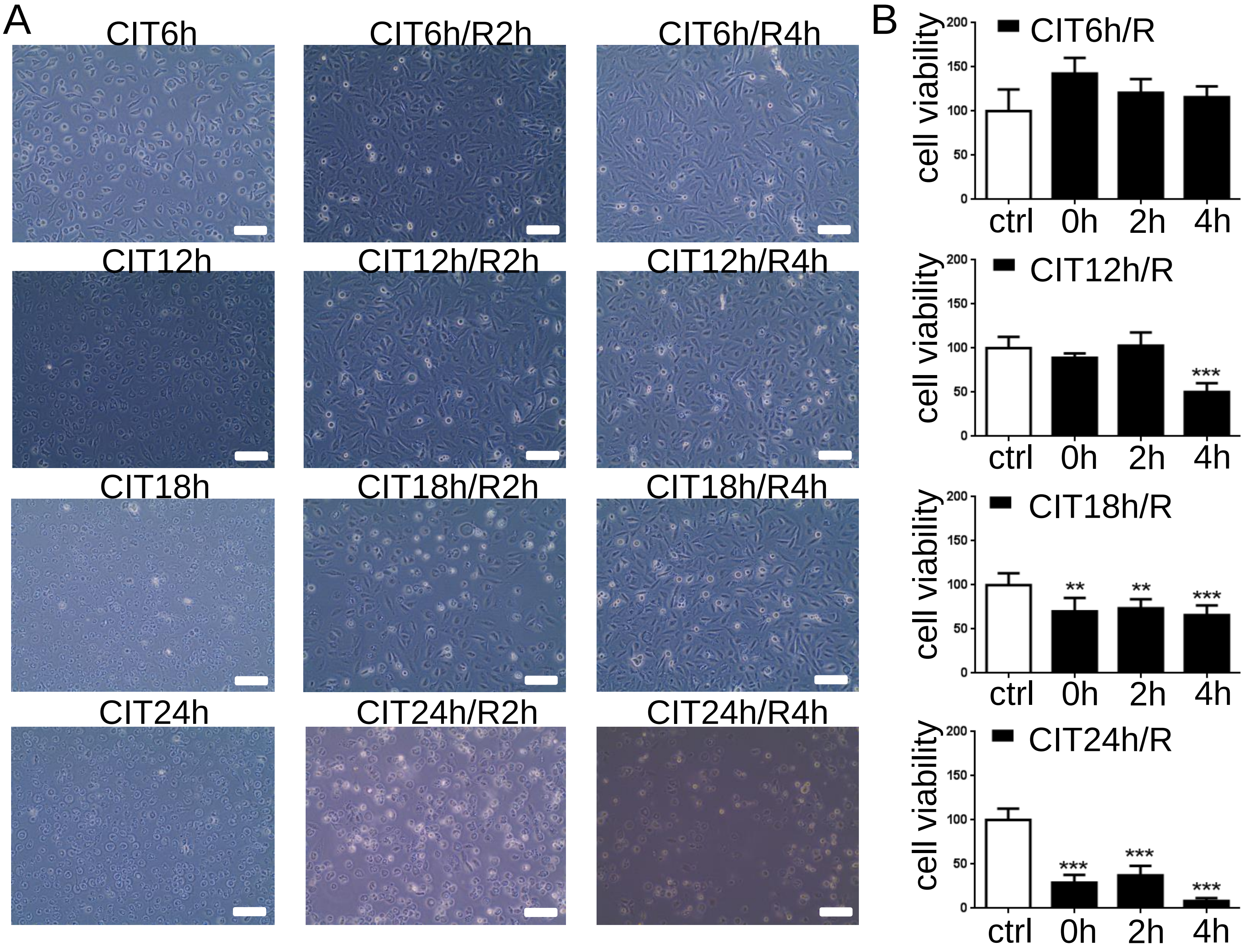

Figure S1. Hypothermic ischemia and normothermic reperfusion induce changes of cell morphology, reduce cell viability in human lung epithelial BEAS-2B cells. (A) BEAS-2B cells underwent 6, 12, 18, or 24 h CIT followed with 2 or 4 h warm reperfusion. A CIT time-dependent change of cell morphology and loss of cells was noted, which was increased after reperfusion (scale bar = 50 μm). (B) The cell viability was quantified via trypan blue exclusion assay. Reduced cell viability was noted after 12 h CIT and reperfusion, which was further enhanced after 18 and 24 h CIT. *P<0.05, **P<0.01, ***P<0.001 vs. control conditions.

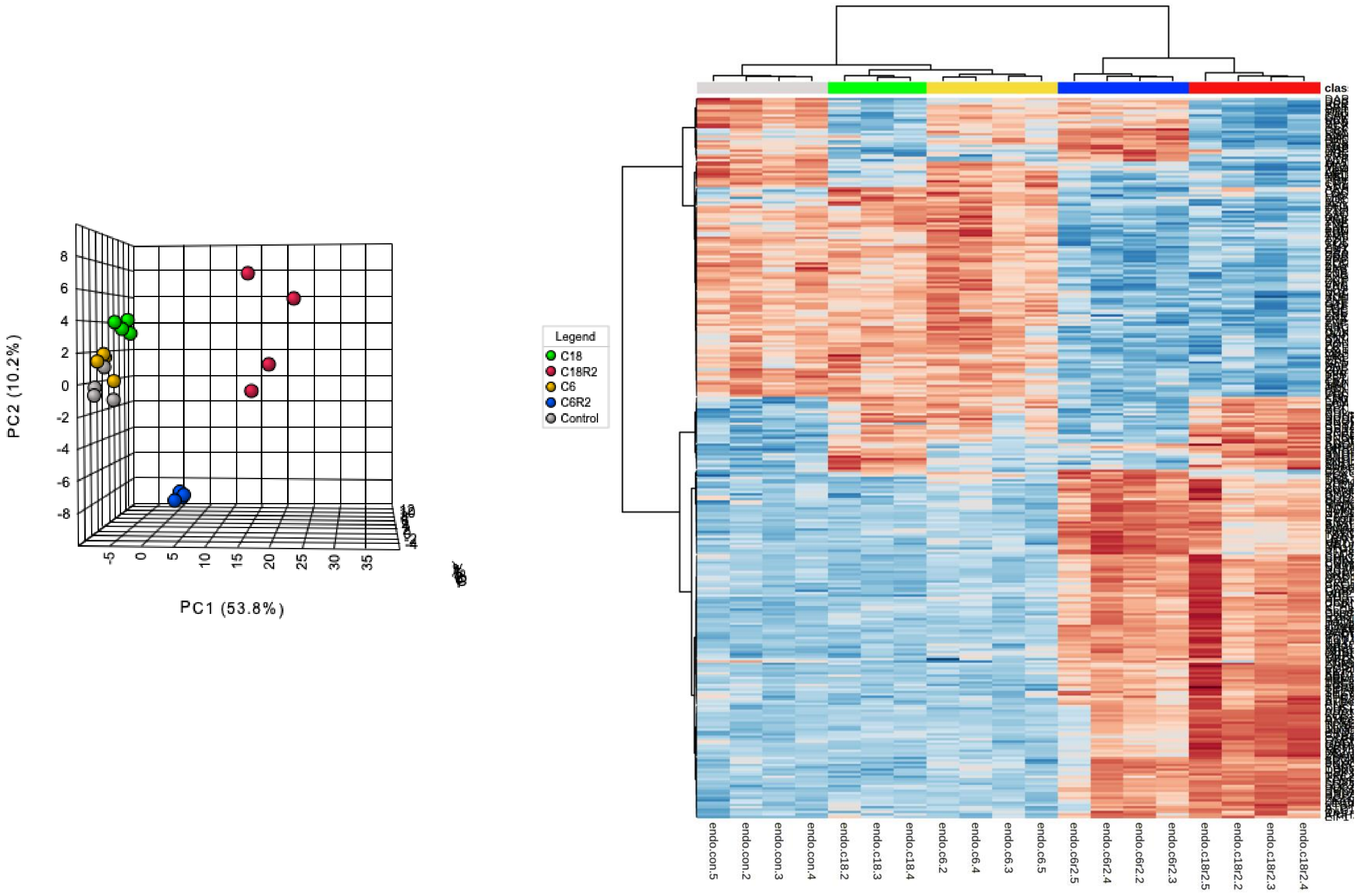

Figure S2. Differentially expressed genes between the five groups within the HPMEC cells with one-way ANOVA and Tukey HSD test. (A) Principal component analysis. (B) Heatmap for 2,901 DE genes with unsupervised clustering.

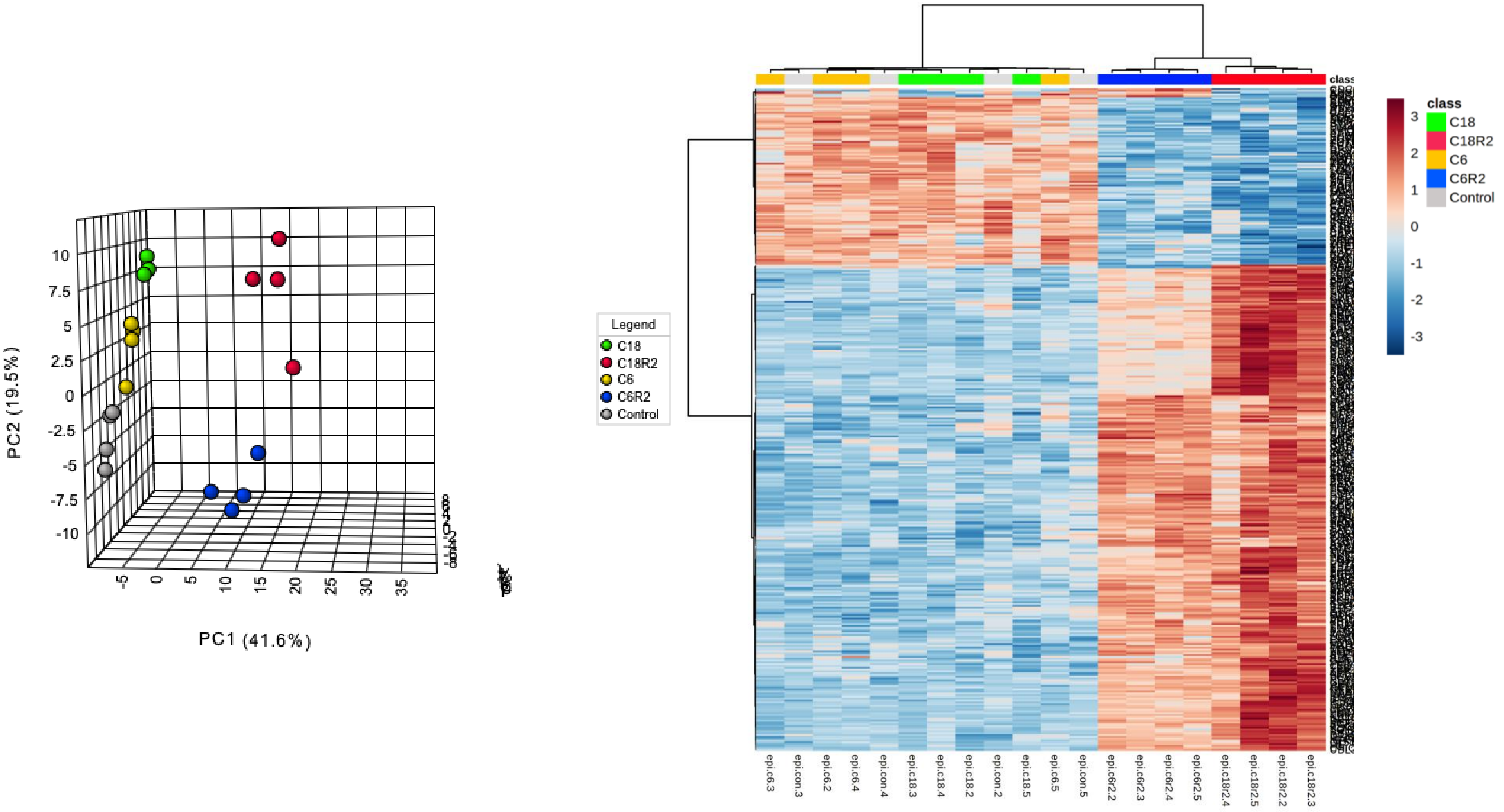

Figure S3. Differentially expressed genes between the five groups within the BEAS-2B cells with one-way ANOVA and Tukey HSD test. (A) Principal component analysis. (B) Heatmap for 2,957 DE genes with unsupervised clustering.

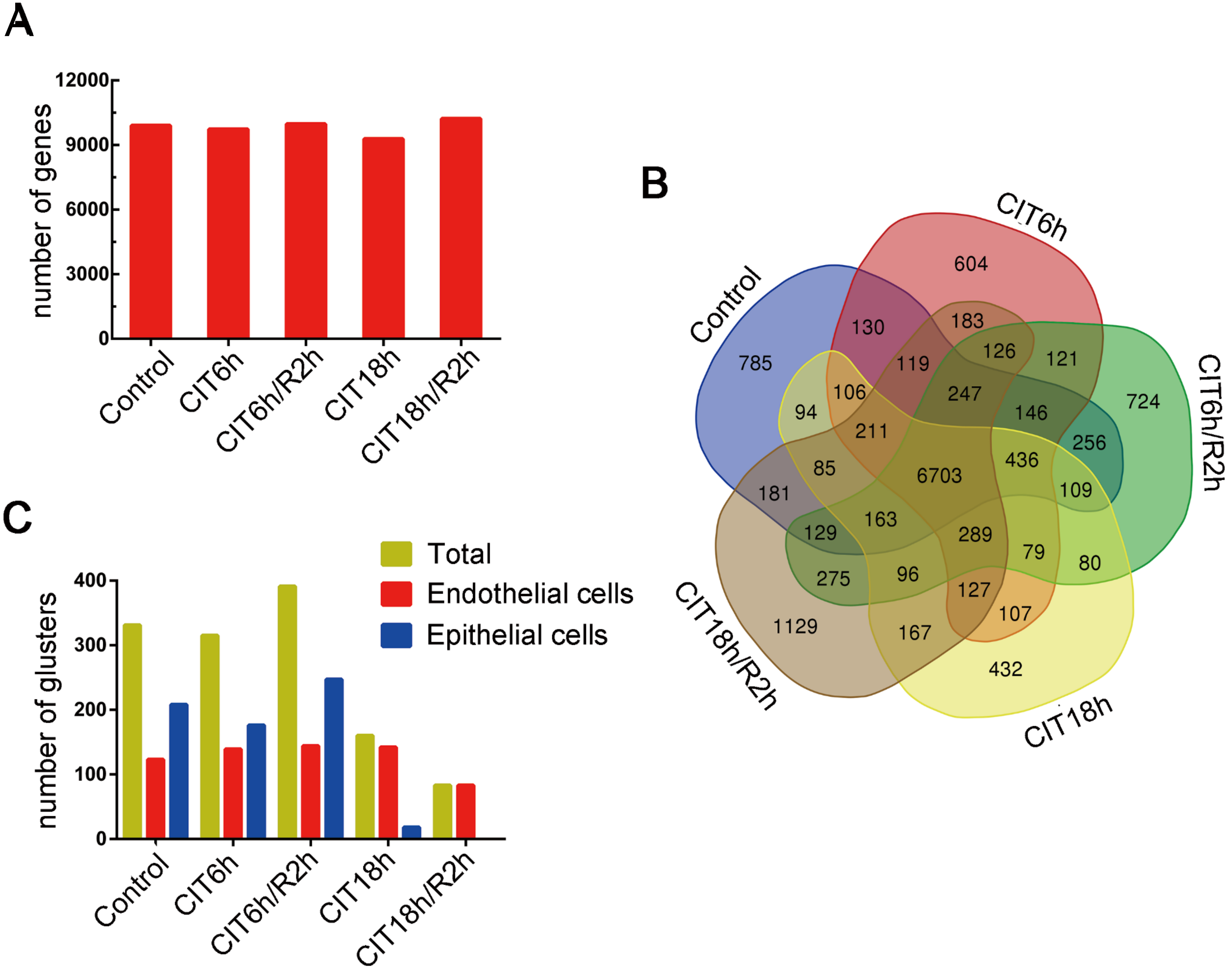

Figure S4. Prolonged cold preservation and reperfusion reduced enriched DE gene-sets. (A) The numbers of DE genes between two cell types remained at the similar levels under different conditions. (B) Venn diagram showed that most of the DE genes are common in all groups, and each group has small number of unique DE genes. (C) The GSEA assay showed that after 18 h CIT, the number of DE gene clusters in epithelial cells was dramatically reduced and disappeared after reperfusion; the number of DE gene-sets in endothelial cells was also reduced.

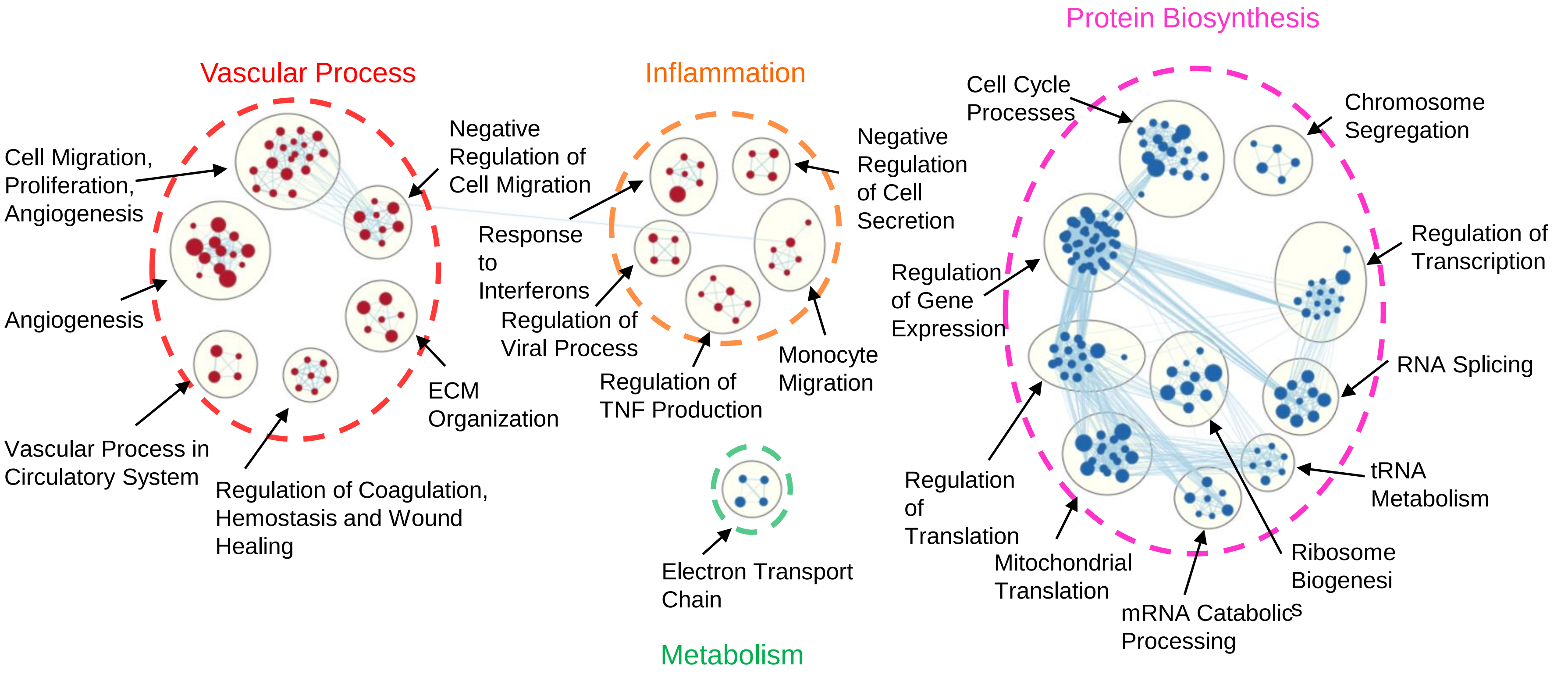

Figure S5. In comparison with control, CIT 6 h did not significantly affect the enriched DE gene clusters between endothelial and epithelial cells. The cut-off of enriched DE gene-sets is FDR<0.05. Clusters enriched in endothelial cells are shown in red nodes, and blue nodes are enriched in epithelial cells.

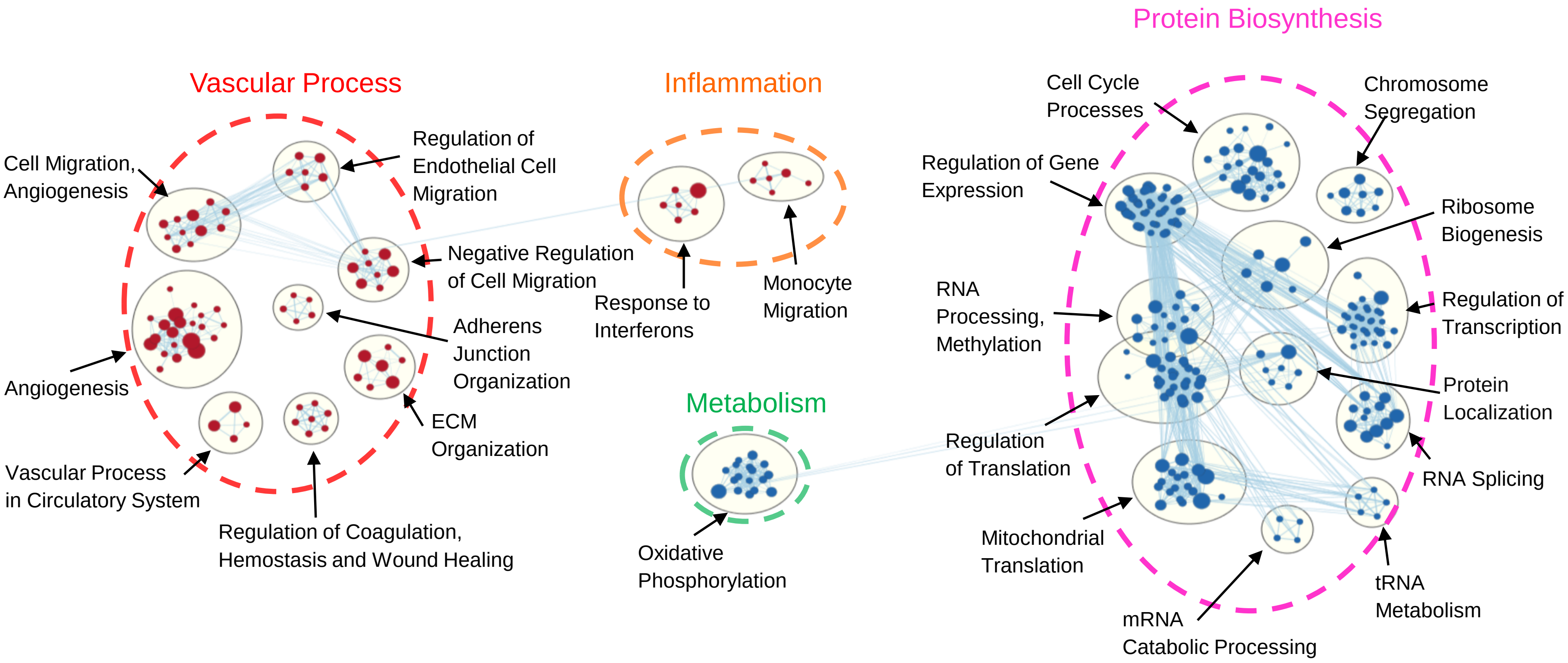

Figure S6. In comparison with control, CIT 6 h followed by 2 h reperfusion also did not significantly affect the enriched DE gene clusters between endothelial and epithelial cells. The cut-off of enriched DE gene-sets is FDR<0.05. Clusters enriched in endothelial cells are shown in red nodes, and blue nodes are enriched in epithelial cells.
